# Mechanism of Hsp70 specialized interactions in protein translocation and the unfolded protein response

**DOI:** 10.1098/rsob.200089

**Published:** 2020-08-19

**Authors:** Natacha Larburu, Christopher J. Adams, Chao-Sheng Chen, Piotr R. Nowak, Maruf M. U. Ali

**Affiliations:** Department of Life Sciences, Sir Ernst Chain Building, Imperial College London, London SW7 2AZ, UK

**Keywords:** Hsp70 chaperones, protein translocation, UPR, BiP, IRE1, Tim44

## Abstract

Hsp70 chaperones interact with substrate proteins in a coordinated fashion that is regulated by nucleotides and enhanced by assisting cochaperones. There are numerous homologues and isoforms of Hsp70 that participate in a wide variety of cellular functions. This diversity can facilitate adaption or specialization based on particular biological activity and location within the cell. In this review, we highlight two specialized binding partner proteins, Tim44 and IRE1, that interact with Hsp70 at the membrane in order to serve their respective roles in protein translocation and unfolded protein response signalling. Recent mechanistic data suggest analogy in the way the two Hsp70 homologues (BiP and mtHsp70) can bind and release from IRE1 and Tim44 upon substrate engagement. These shared mechanistic features may underlie how Hsp70 interacts with specialized binding partners and may extend our understanding of the mechanistic repertoire that Hsp70 chaperones possess.

## 1. Introduction

Hsp70 chaperones are critical components of processes that maintain the integrity of proteins within the cell. They are involved in a wide variety of cellular activities that includes folding of newly synthesized proteins; translocation of nascent polypeptides into organelles such as mitochondria, endoplasmic reticulum (ER) and chloroplasts; disassembly of protein complexes and activation of various proteins. Furthermore, in both stressful and non-stressful conditions, they prevent the aggregation of misfolded proteins and coordinate their refolding enabling them to revert to their native state [1–3]. Because of its central role in regulating protein homeostasis, any aberrations in its function will have a major impact on the ability of the cell to maintain protein integrity. This can lead to an accumulation of misfolded proteins or non-native intermediate forms of proteins. Such situations are deleterious for cell fitness and may result in diseases that are associated with protein aggregates including Alzheimer's and cellular ageing [1–3].

Several decades of research have carefully pieced together the mechanism of how Hsp70 chaperones interact with their substrates, a process that is tightly regulated by nucleotides and enhanced by helper proteins known as cochaperones. There are a considerable number of Hsp70 homologues and isoforms present in nature and an even greater variety of assisting cochaperones. This complexity can be explained by the range of biological roles that Hsp70 chaperones are implicated in. Also, this diversity facilitates a certain level of adaption based on cellular location and particular cellular function, and is often referred to as specialization [2,4].

The role of Hsp70 in driving protein translocation across the inner mitochondrial membrane is a well-described example of how Hsp70 can specialize to fulfil an essential cellular activity. The ER equivalent homologue, known as BiP (binding immunoglobulin protein), can also operate at the membrane to facilitate protein translocation into the ER lumen. Moreover, BiP has been suggested to play a role in the unfolded protein response (UPR), an essential homeostasis pathway that detects and responds to an increase of misfolded protein within the ER [5–7]. How it operates in this capacity is much less clear and subject to some debate. In this review, we highlight some recent mechanistic advances into BiP UPR activity which suggests some clear analogy with the actions of mitochondrial Hsp70 (mtHsp70) during protein translocation. These similarities indicate common mechanistic features that may underlie how Hsp70 chaperones interact with specialized binding partners.

## 2. Hsp70 chaperone mechanism and substrate interactions

**Figure 1.**
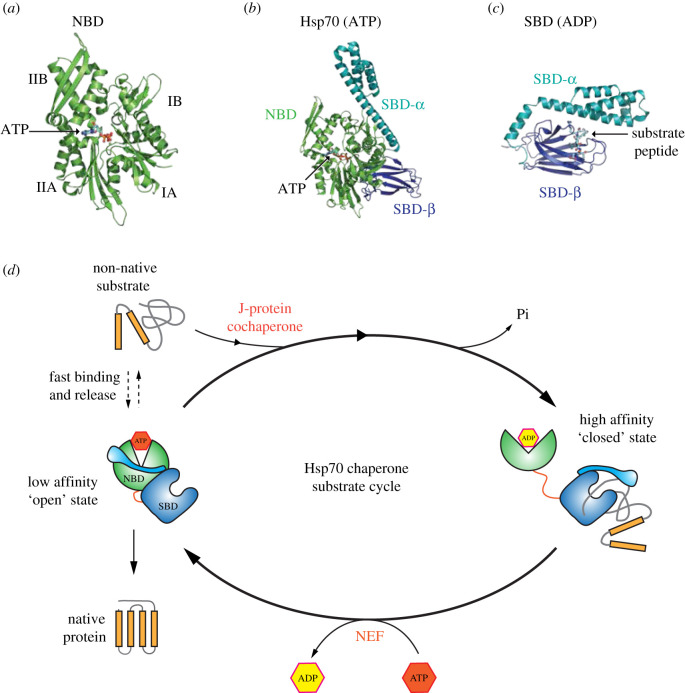
Hsp70 chaperone substrate cycle. (*a*) Cartoon representation of Hsp70 NBD showing the organization of the four subdomains IA, IB, IIA, IIB, with ATP bound in the active site (PDB 4B9Q [9]). (*b*) The full-length structure of Hsp70 bound to ATP, illustrating the arrangement of the two domains NBD and SBD relative to one another. (*c*) The structure of SBD bound to substrate peptide [14]. The SBD*α* lid closes over the peptide binding site, which is present within the SBDβ subdomain, trapping the substrate. (*d*) A schematic diagram depicting Hsp70 conformations as regulated by nucleotides. When Hsp70 is bound to ATP, there is close association between NBD and SBD that results in the lid (SBDα) being positioned such that it exposes the peptide binding site within SBDβ. This favours fast substrate binding and release and works in synergy with J-protein cochaperone that facilitates substrates recruitment to Hsp70. The association of J-protein with substrate mediates ATP hydrolysis, which enables Hsp70 to transition to the closed conformation. In this state, the SBDα closes over the substrate. NEF mediates the exchange of ADP to ATP thus facilitating the transition to the open ATP-Hsp70 bound conformation, where substrate release can occur.

Hsp70 chaperones undergo coordinated movements that facilitate misfolded substrate binding and release. The domain architecture of Hsp70 is generally well conserved across different species and homologues. It comprises a nucleotide-binding domain (NBD) and a substrate-binding domain (SBD) that is connected via a short linker. The NBD is made up of four subdomains that are organized into two lobes which results in the formation of a deep cleft [8,9] (figure 1). The active site—the site where ATP binding and hydrolysis takes place—is situated at the base of the cleft. ATP makes contact with all four subdomains and can facilitate subtle movements throughout the NBD. Typically, the rate of ATP hydrolysis is low with a turnover of 1 ATP molecule every 20–30 min [8,10–12]. The SBD interacts with the substrate protein by engaging a short motif within the target polypeptide. This sequence usually consists of five hydrophobic amino acids that are flanked by charged residues [13]. This type of motif is present in nearly all proteins, often occurring multiple times, and enables a great versatility in substrate selection. The SBD is further divided into two subdomains, SBD*α* and SBDβ (figure 1*b,c*). The SBDβ consists of an eight-stranded β-sandwich fold which contains the hydrophobic pocket that constitutes the polypeptide-binding site. The SBD*α* is a helical structure that acts as a lid which can close over the polypeptide-binding pocket [14,15].

The binding and release of the substrate is allosterically coupled to nucleotide association and ATP hydrolysis. In the ATP bound state, Hsp70 interacts with substrate protein with a high *K*_on_ and *K*_off_ (low affinity) rate enabling the loading and release of the substrate. Upon ATP hydrolysis and transition to the ADP bound state, Hsp70 undergoes structural re-arrangements that include the positioning of the SBD*α* lid over the SBD-binding pocket, with the effect of trapping the substrate within the SBD commensurate with a much reduced *K*_on_ and *K*_off_ rate (high affinity) [3,12,16–19] (figure 1*d*). There are two groups of Hsp70 cochaperones that facilitate the transition between states. (i) J-domain containing proteins that increases hydrolysis thereby stabilizing the substrate interaction. (ii) Nucleotide exchange factors (NEF) that mediate the exchange of ADP to ATP enabling the release of the bound substrate [16,17] (figure 1*d*). Once maximal ATP hydrolysis is achieved, the effective affinity for the substrate increases by several orders of magnitude in a non-equilibrium fashion and is referred to as ultra-affinity [20]. Thus, it is the concerted activity of the cochaperones, along with substrate binding, that stimulates the low efficiency of Hsp70 ATP hydrolysis.

## 3. The role of Hsp70 in protein translocation at the endoplasmic reticulum membrane

**Figure 2.**
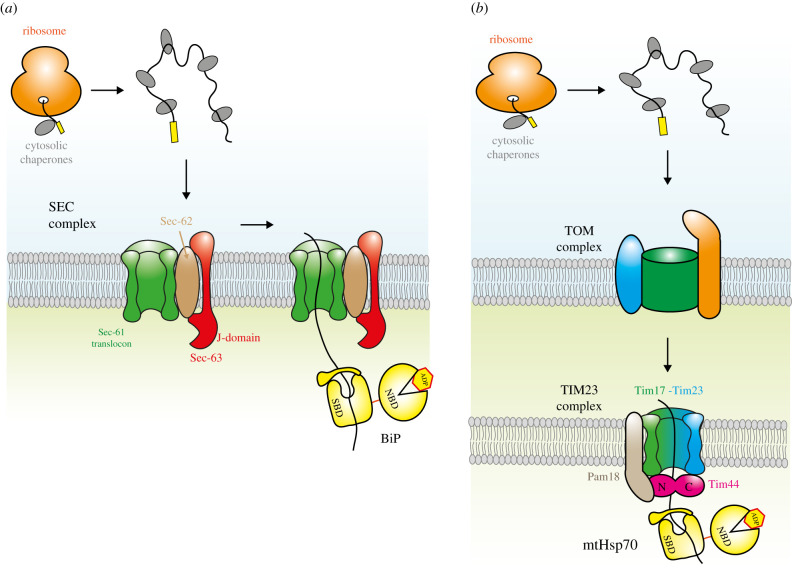
Protein translocation. (*a*) Diagram depicting post-translational translocation into the ER. The polypeptide chain is fully synthesized and dissociated from the ribosome but kept in a partially folded state by cytosolic chaperones (not shown to scale). The nascent chain interacts with the membrane bound Sec61-62-63 complex which facilitates its translocation into ER lumen. The driving force for polypeptide insertion comes from BiP. Initially, BiP is attached to Sec63. As the polypeptide chain translocates through the channel, BiP binds to the nascent chain causing its release from Sec63. This serves to increase its entropy as it moves away from the constraints of the membrane. (*b*) Protein translocation into the mitochondrial matrix. A simplified diagram illustrating the pre-sequence pathway for protein insertion in the matrix. Newly synthesized polypeptides attach to cytosolic chaperones that interact firstly with the TOM translocase complex and subsequently with TIM23 complex. Integral membrane proteins usually engage a different translocase complex within the inner membrane (TIM22) (not shown). Also, TIM23 complex can transfer proteins laterally into the membrane depending on certain cues within the polypeptide sequence. The PAM motor helps drive the pre-sequence polypeptide into the matrix by entropic pulling of the nascent chain by mtHsp70. The key PAM components, mtHsp70 (yellow), Tim44 (pink) and Pam18 (light brown) are shown connected to the channel forming components Tim17-Tim23 (green - blue).

As newly synthesized polypeptides are translated by the ribosome within the cytosol, their N-terminal hydrophobic signal sequence is recognized by signal recognition particle which then attaches to it [21]. The association of the SRP directs the nascent chain–ribosome complex to SRP receptor located at the ER membrane to stimulate GTP hydrolysis [22]. This causes the release of SRP from the nascent chain and the SRP receptor, which in turn mediates the transfer of the complex to a protein conducting channel known as Sec61 translocon, and is coincident with the resumption of protein synthesis by the attached ribosome [23]. As the ribosome translates the nascent chain, it inserts the newly synthesized polypeptide into the ER via the Sec61 translocon in a process termed co-translational translocation. In some situations, the signal sequence is not recognized and the translating nascent chain associates with cytosolic Hsp70 along with its cochaperone Hsp40 instead of capture by SRP. This interaction enables the nascent protein with its bound chaperones to engage an integral ER membrane complex that consists of Sec61 conducting channel and two further membrane partner proteins termed Sec62 and Sec63 [24,25]. Sec63 contains a J-domain on its luminal side, which can interact with the ER Hsp70 chaperone, BiP. As the nascent protein is threaded through the conducting channel, BiP binds the polypeptide once it enters into the ER lumen and helps to drive the rest of the protein through the channel. Furthermore, this association prevents aggregation and enables the correct folding of the nascent chain. In this process, the polypeptide chain is already synthesized by the ribosome before it is brought to the conducting channel for insertion into the ER and is known as post-translational translocation [24,25] (figure 2).

## 4. The role of Hsp70 in protein translocation into mitochondrial matrix

Post-translational translocation is the typical method for delivery of nascent proteins into the mitochondrial matrix. Newly synthesized proteins can contain a precursor sequence that is hydrophobic and positively charged. After translation by the ribosome, the nascent chain interacts with cytosolic Hsp70 chaperones. This prevents the protein from fully adopting its native folded structure and facilitates its passage through the conducting channel located in both the outer and inner mitochondrial membranes. The outer membrane translocation machinery is composed of the conducting channel proteins Tom40 and two associated receptor proteins Tom70 and Tom20. The receptor proteins engage the Hsp70 chaperone-nascent chain complex, which enables the translocation of the polypeptide through the Tom40 pore where it associates with the inner membrane conducting complex, TIM23. The TIM23 complex comprises the essential inner membrane pore-forming proteins Tim17 and Tim23, and the membrane protein Tim50. The negative potential across the inner membrane combined with the action of a dedicated motor, known as the pre-sequence mitochondrial associated motor or PAM, situated on the matrix side of the TIM23 complex, helps to drive the positively charged pre-sequence through the channel and into the matrix [4,26] (figure 2).

The PAM motor complex is made up of five essential subunits in yeast. Three of these subunits are a part of the Hsp70 chaperone system, including mtHsp70 (Ssc1), NEF Mge1 and the membrane-spanning J-protein Pam18 [27]. mtHsp70 along with Mge1 are involved in other processes alongside their participation in PAM motor activities and serve as major factors in general protein folding. In this role, mtHsp70 interacts with different J-proteins and not Pam18, which is specific for protein translocation. The action of Pam18 is similar to the ER homologue Sec63, both of which are membrane-spanning J-proteins involved exclusively in protein translocation across membranes.

The other two essential components of PAM are Tim16 and Tim44, they both act as adaptor proteins that have multiple contacts with the other PAM components. Tim16 contains a degenerate J-like domain which is unable to stimulate Hsp70 activity. It engages Pam18, an association that is important for Pam18 to interact with the conducting channel protein, Tim23 [28,29].

Tim44 serves as a central hub protein that crucially links the conducting channel to PAM motor proteins on the matrix side of the pore. Tim44 is divided into two domains of approximately equal size and referred to as the N- and C-terminal domains [30,31]. The NTD primarily contacts Hsp70 and Pam16, while the CTD interacts with Tim23 [4,29,32].

During translocation of the pre-sequence through the TIM23 pore, the emerging polypeptide engages mtHsp70. This association releases mtHsp70 from Tim44 and in the process helps to drive through the nascent chain into the matrix. When mtHsp70 is attached to Tim44, it has limited movement due to the constraints of being close to the membrane. The binding of the partially folded pre-sequence polypeptide frees mtHsp70 from its location-based constraints, increasing its entropy, which provides the driving force for translocation [2,4,26] (figure 2).

## 5. The role of BiP in unfolded protein response signalling

The UPR is a cell signalling system that detects the presence of misfolded proteins within the ER and initiates a transcriptional and translational response that aims to restore ER homeostasis. IRE1 is a key primary activator/sensor of the UPR that is conserved from yeast to humans. It spans the ER membrane presenting both luminal and cytosolic domains [5–7]. The luminal domain (LD) senses misfolded proteins within the ER, either directly or via BiP, which leads to activation of its cytosolic domain. There are two enzymatic reactions that are mediated by the cytosolic portion of IRE1: phosphorylation and endoribonuclease splicing. Upon activation, the kinase subdomain of one IRE1 monomer is responsible for auto-phosphorylating the opposing monomer when IRE1 is arranged in a dimer formation [33–36]. The extreme C-terminus of IRE1 comprises the endonuclease subdomain, which specifically cleaves Xbp1 mRNA. The spliced form of Xbp1 (sXbp1) codes for a potent transcriptional activator that upregulates UPR target genes [37,38]. IRE1 stimulation can also lead to indiscriminate splicing that causes mRNA decay at the ER membrane in a process termed regulated IRE1-dependent decay (RIDD) [39].

**Figure 3.**
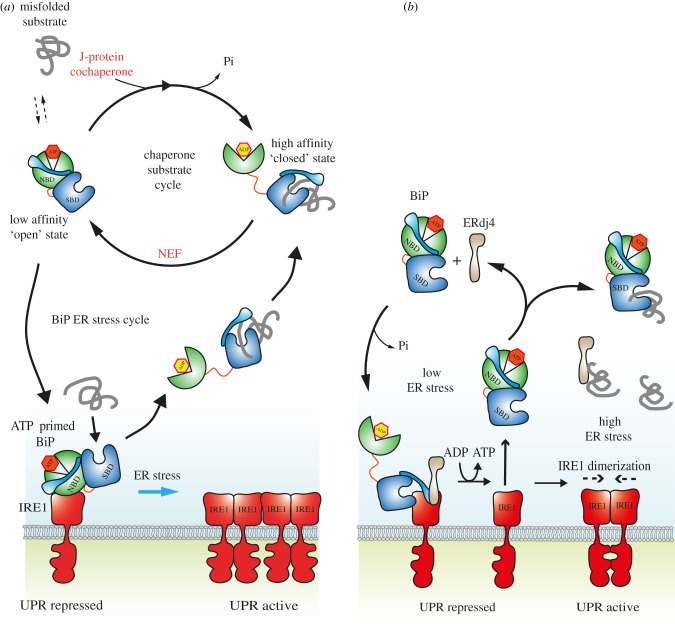
BiP-dependent UPR activation models. (*a*) Allosteric model for ER stress sensing. BiP interacts with IRE1 via its NBD domain, preventing BiP association with its cochaperones, which switches BiP to an ER stress sensor role. ATP primes BiP to engage misfolded proteins. The binding of misfolded protein to BiP SDB triggers release of BiP NBD from IRE1 via a conformational change. IRE1 dimerizes or oligomerizes to activate UPR signalling. BiP is now able to associate with its cochaperones and refold the attached misfolded protein via its well characterized nucleotide dependent substrate cycle. (*b*) Competition model for UPR repression. BiP binds to IRE1 via its SBD as a chaperone substrate interaction. This is the same site that misfolded proteins bind to BiP. ERdj4 is required to recruit BiP to an IRE1 dimer in order to break the dimer and repress UPR signalling in a process that requires ATP hydrolysis. The ADP bound form of BiP causes release of ERdj4 from IRE1-BiP complex. Nucleotide exchange factors enable the exchange of ADP to ATP bound BiP. The ATP bound form of BiP results in the dissociation of BiP from IRE1 monomer. Now, the free IRE1 monomer can either spontaneously form an IRE1 dimer leading to UPR activation, or BiP could rebind IRE1 via ERdj4 active targeted recruitment and keep UPR repressed. In high ER stress, BiP and ERdj4 are occupied binding to misfolded protein so the number of ATP bound BiP and ERdj4 is sufficiently low that there is none available to rebind IRE1 leading to UPR activation. In low ER stress, there is a preponderance of BiP and ERdj4 enabling UPR repression.

BiP has been suggested to play a role in UPR signalling, although precisely what that may be is subject to debate and contingent upon the various models that have been proposed. A BiP independent UPR activation model suggests that IRE1 directly contacts misfolded proteins, with BiP playing a peripheral role in sequestering inactive IRE1 [40,41]. By contrast, a BiP-dependent ‘competition' model postulates that IRE1 and misfolded proteins compete for binding to polypeptide-binding site situated in BiP SBD. In low ER stress conditions, BiP attachment to IRE1 will repress UPR signalling analogous to heat shock factor 1 (Hsf1) repression by cytosolic Hsp70 [42]. In this model, IRE1 acts as a substrate protein, the interaction of which is regulated by ATP and J-protein cochaperone, ERdj4 [43,44]. An alternative BiP-dependent ‘allosteric’ model proposes that binding of IRE1 to BiP occurs via NBD, and that dissociation between the two proteins is dependent on misfolded protein engaging BiP SBD, which lowers the affinity of BiP NDB for IRE1 [7]. In this model, BiP acts by detecting misfolded protein, which leads to UPR induction [7]. The two modes of binding between IRE1 and BiP (either via BiP NBD or SBD) give rise to significantly different models with contrasting mechanistic implications (figure 3). However, both of these models may occur at different time points, thus explaining both sets of observations. The more established BiP ‘competition' model based on Hsf1 repression mechanism has been extensively reviewed in Pressler and Ron 2018 [43]. In this article, we focus on BiP NBD interaction with IRE1 as part of the ‘allosteric' UPR sensing/induction model [7].

## 6. Hsp70 specialized interactions at the membrane

The aforementioned examples of Tim44 and IRE1 are two instances where Hsp70 associates with proteins that are neither a cochaperone (i.e. they do not contain a J-protein), or have NEF ability, or are a misfolded substrate protein. Such intriguing interactions arise due to specialization imposed by the cellular location and the requirement to fulfil a specific biological role. Interestingly, recent data suggests analogy between the well-defined actions of mtHsp70 when binding to Tim44 with that of BiP association to IRE1 in the ‘allosteric' UPR model. These mechanistic similarities are discussed below.

First, both BiP and mtHsp70 chaperones are adapted to operate at the membrane due to their ability to bind membrane-spanning or membrane-associated proteins. This enables them to operate in protein translocation and UPR signal induction, as both of these processes occur at the membrane [4,6,7,26]. Besides this, both chaperones have major roles in other activities including general protein folding and assembly. In order to fulfil multiple roles simultaneously, they are highly expressed in comparison to their specialized binding partner and are often one of the most abundant proteins within the organelle. For example, there are roughly 2500–5000 copies of IRE1 in HeLa cells [45], while BiP numbers are usually greater than 2 × 10^7^ [46]. This disparity in concentration may create an equilibrium that could favour association between specialized partner proteins and chaperones as a default state. This would have minimal effect on the total number of Hsp70 that could take part in general protein folding or other processes at the same time.

**Figure 4.**
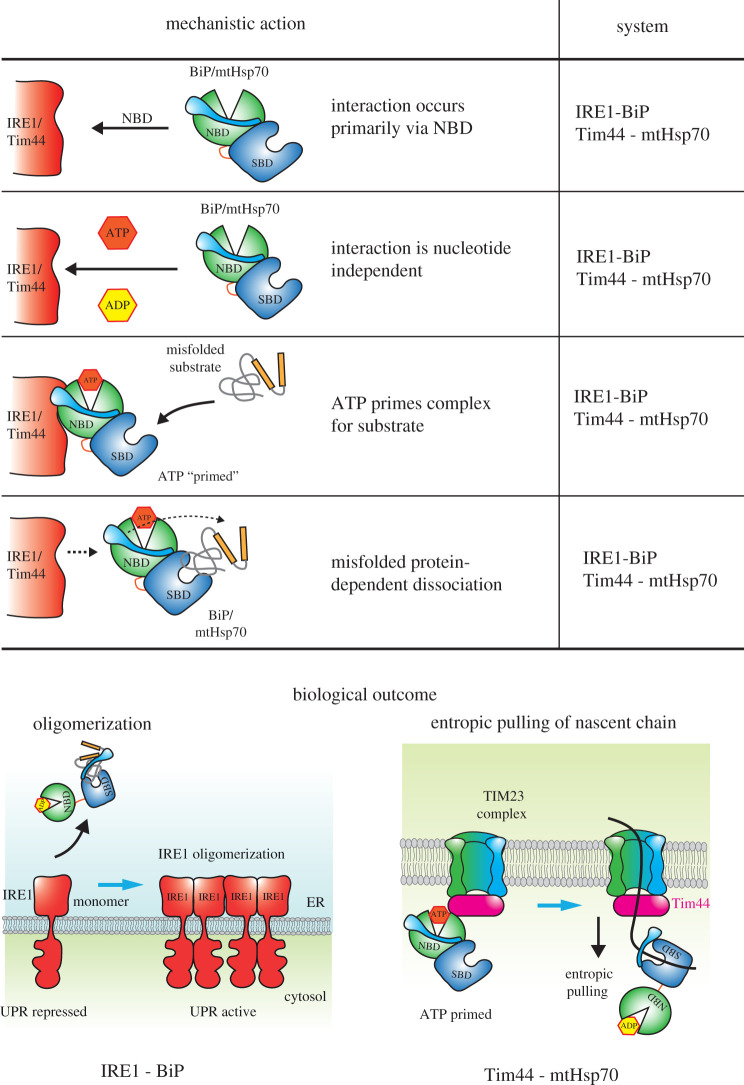
A graphical summary illustrating the key mechanistic features of specialized interactions involved in IRE1-BiP and Tim44-mtHsp70 systems, and their differing biological outputs.

Second, the interaction with Tim44 and IRE1 is primarily mediated by the NBD of mtHsp70 and BiP, respectively (figure 4), although, for Tim44, the NBD interaction may be supplemented by contributions from the SBD, but not as a substrate [47]. Competition experiments indicated that peptide binding to mtHsp70 was unaltered on addition of Tim44 suggesting that Tim44 itself does not serve as a substrate or interfere with peptide binding [47]. Similarly, other studies have indicated that binding to Tim44 occurs directly via the NBD [48,49].

There are several lines of evidence to suggest that IRE1 interacts primarily via BiP NBD. This includes biophysical experiments that measure a direct interaction between BiP NBD and IRE1 LD using microscale thermophoresis [50]. This is further complimented by pull-down interaction analysis and chemical cross-linking that similarly observe a direct interaction with BiP NBD [50]. A recent structure-guided mutational study identified a mutation within the NBD that severely reduced the affinity for binding to IRE1 [51]. Furthermore, in another mutational-based study that was conducted in yeast cells, it was suggested that the interaction with IRE1 was again mediated via BiP NBD [52].

Third, the interaction between Hsp70 and specialized binding partners, Tim44 and UPR proteins, are not destabilized by nucleotides. Fourth, that dissociation between specialized binding partners and Hsp70 is dependent on misfolded protein substrate binding and this process is primed by ATP (figure 4). Both of these points are linked. Initial co-immunoprecipitation experiments performed in cells suggested that Tim44 interaction to mtHsp70 was dissociated by addition of ATP [48,53]. However careful analysis using purified proteins demonstrated that the interaction was stable both in the presence of ADP and ATP. It was only the addition of substrate peptide that caused dissociation of Hsp70 from Tim44 *in vitro*. In cells, there are numerous partially folded proteins that can act as substrate and the addition of ATP was suggested to prime the engagement of misfolded protein leading to dissociation of mtHsp70 from Tim44 [47,54]. Similarly, for IRE1, co-immunoprecipitation experiments suggested that BiP was dissociated from IRE1 on the addition of ATP [55,56]. However, reconstitution experiments using purified proteins demonstrated that BiP was able to form a complex with IRE1 in the presence of both ATP and ADP [50]. Significantly, the dissociation was dependent on misfolded protein binding to the SBD of BiP, which in turn elicited the release of BiP NBD from IRE1 [50]. This was conspicuously demonstrated using a three-way *in vitro* FRET assay. In these experiments, IRE1 LD and BiP were N-terminally tagged with cyan and yellow fluorescence proteins, which on mixing produced a FRET signal. The subsequent addition of misfolded proteins, but not nucleotides, caused the loss of FRET signal, indicating again the misfolded substrate-dependent dissociation of BiP from IRE1 [57]. Moreover, a mutation situated in the polypeptide-binding site that renders BiP unable to associate with the substrate (BiP V461F), prevented the dissociation of the complex in the presence of misfolded protein [57]. This indicates that the release of BiP from IRE1 requires the binding of misfolded protein to BiP SBD and not to IRE1 LD. IRE1 LD itself has been shown to interact with misfolded protein [41], although this association may occur after the release of BiP from IRE1 [40].

Additional *in vitro* FRET experiments, measuring the influence of nucleotides on substrate-dependent dissociation of this complex, indicated a clear priming effect in the presence of ATP, but not ADP [51]. The release of BiP from IRE1 in the presence of ATP requires 21-fold less misfolded substrate than in the ADP bound state. Thus, the addition of ATP primes the IRE1-BiP complex for interaction with misfolded proteins [51]. This offers an explanation to the earlier observation that ATP destabilizes the complexes isolated from cells, with a similar rationale to mtHsp70 dissociation from Tim44.

For BiP, the binding of IRE1 prevents the association of its cochaperones, ERdj3 and Sil1. ERdj3 and Sil1 are the canonical cochaperones for BiP when performing general protein-folding activities within the ER. This in effect switches BiP from acting as a chaperone to being fully primed to detect misfolded protein. Once BiP binds substrate, it detaches from IRE1 enabling ERdj3 (or Sil1) to interact with BiP to mediate its chaperone substrate cycle [51]. This suggests that the specialized binding partner is able to co-opt BiP to serve in UPR signal induction, making use of the fact that it can efficiently bind misfolded proteins. The release of BiP from IRE1 may facilitate a dimerization/oligomerization event that leads to further UPR signal propagation, primarily by activating its kinase trans-phosphorylation reaction [7]. Thus far, it's not known whether the binding of Tim44 prevents the association of the canonical cochaperone Mjd1 to mtHsp70.

## 7. Conclusion

In this review, we highlight two Hsp70 homologues that have specialized roles in protein translocation and stress signalling at the membrane. In their orthodox activities, which include protein folding and prevention of aggregation, their interaction with substrate protein is well characterized and involves the coordinated movement of their two domains upon ATP binding and hydrolysis, a process that is catalysed by cochaperones. The iterative cycling between such conformations leads to a non-equilibrium high-affinity state that mediates the remodelling of the substrate to help form its native structure. In their alternative specialized roles, they can interact with proteins that are neither cochaperones nor substrates. This type of interaction suggests additional mechanistic features beyond that which is required for interaction with misfolded substrates. In this capacity, we highlight some common features of two specialized binding partner proteins, Tim44 and IRE1, when they interact with their complimentary Hsp70 chaperones, mtHsp70 and BiP. Such features include binding that is mediated primarily via the NBD. Nucleotides do not affect this association but exert their influence by priming the SBD to engage substrate protein. A key defining feature seems to be that dissociation of specialized partner from Hsp70 is dependent on misfolded substrate binding to SBD. The consequences of substrate-dependent dissociation are different for the two homologues as they serve different biological roles. Tim44 presents a platform for mtHsp70 that enables it to bind the nascent translocating polypeptide chain. Subsequent release from the confines of the membrane increases the entropy of the system and provides the driving force for polypeptide insertion into the matrix. For BiP, misfolded-dependent dissociation would presumably trigger oligomerization leading to downstream activation and propagation of the UPR signalling cascade. It would be interesting to observe whether these common mechanistic features can be extended to other specialized binding partners that are positioned away from the membrane. These features may expand our understanding of the mechanistic repertoire that Hsp70 chaperones possess. Thus, in summary, we suggest common mechanistic features for binding and release of BiP and mtHsp70 with specialized binding partners at the membrane.

## Data accessibility

This article does not contain any additional data.
